# A comprehensive review of BBV152 vaccine development, effectiveness, safety, challenges, and prospects

**DOI:** 10.3389/fimmu.2022.940715

**Published:** 2022-09-13

**Authors:** Farokh Dotiwala, Arun K. Upadhyay

**Affiliations:** Ocugen Inc., Malvern, PA, United States

**Keywords:** SARS – CoV – 2, vaccine, heterologous prime and boost vaccines, inactivated virus adjuvanted vaccine, variants of concern (VOCs), vaccine challenges, original antigenic sin

## Abstract

The world has responded to the COVID-19 pandemic with unprecedented speed and vigor in the mass vaccination campaigns, targeted to reduce COVID-19 severity and mortality, reduce the pressure on the healthcare system, re-open society, and reduction in disease mortality and morbidity. Here we review the preclinical and clinical development of BBV152, a whole virus inactivated vaccine and an important tool in the fight to control this pandemic. BBV152, formulated with a TLR7/8 agonist adjuvant generates a Th1-biased immune response that induces high neutralization efficacy against different SARS-CoV-2 variants of concern and robust long-term memory B- and T-cell responses. With seroconversion rates as high as 98.3% in vaccinated individuals, BBV152 shows 77.8% and 93.4% protection from symptomatic COVID-19 disease and severe symptomatic COVID-19 disease respectively. Studies in pediatric populations show superior immunogenicity (geometric mean titer ratio of 1.76 compared to an adult) with a seroconversion rate of >95%. The reactogenicity and safety profiles were comparable across all pediatric age groups between 2-18 yrs. as in adults. Like most approved vaccines, the BBV152 booster given 6 months after full vaccination, reverses a waning immunity, restores the neutralization efficacy, and shows synergy in a heterologous prime-boost study with about 3-fold or 300% increase in neutralization titers against multiple SARS-CoV-2 variants of concern. Based on the interim Phase III data, BBV152 received full authorization for adults and emergency use authorization for children from ages 6 to 18 years in India. It is also licensed for emergency use in 14 countries globally. Over 313 million vaccine doses have already been administered in India alone by April 18^th^, 2022.

## Introduction

Severe acute respiratory syndrome coronavirus 2 (SARS-CoV-2) has created a global emergency due to the rapid worldwide spread of coronavirus disease 2019 (COVID-19) (1). As of July 26, 2022, SARS-CoV-2 and its variants have caused more than 571 million infections and 6.38 million deaths, with the US alone accounting for 89.6 million cases and 1,018,073 deaths (covid19.who.int). In earlier SARS-CoV-2 variants about 15% of infected patients developed pneumonia and around 5% of patients developed more critical symptoms such as acute respiratory distress syndrome and multiple organ failure (2, 3). The Omicron variant (B.1.1.529) while being much more (2-5-fold) infective than the Delta (B.1.617.2) variant shows 2-5-fold less fatality (4), and caused significantly less morbidity, hospital admissions and requirement for oxygen supplementation in adults (5). However, the hospitalization rates for children were higher during the Omicron peak than the Delta peak and might be explained by a shift in the COVID-19 symptoms due to Omicron. Severe forms of COVID-19 are associated with lymphocyte dysfunction, monocyte and granulocyte abnormalities leading to cytokine storm with increased levels of IL-1β, IL-6, IL-2, IL-8, IL-17, IP10, MCP1, MIP1α, G-CSF, GM-CSF, and TNF-α. High levels of C-reactive protein (CRP), D-dimer, immunoglobulin G (IgG), and total antibodies are also observed in severe COVID-19 (6–9). However, reports investigating the correlation between high viral loads and COVID-19 severity vary from high to no statistical correlation (10–19).

More than 6.5 million globally surveilled SARS-CoV-2 sequences shared by the Global Initiative on Sharing All Influenza Data (GISAID), allow real-time tracking of SARS-CoV-2 mutations and new variants (20). While many of the SARS-CoV-2 mutations are expected to be either neutral or deleterious for the virus, a small proportion of these mutations alter the virulence, transmissibility, and viral interactions with host immunity, which confer a survival advantage to the virus. Most of these beneficial mutations have occurred in key areas of the SARS- CoV-2 immunodominant spike (S) protein leading to the emergence of five main variants of concern (VOC): Alpha (B.1.1.7) in the United Kingdom, Beta (B.1.351) in South Africa, Gamma (B1.1.28-P.1) in Brazil, Delta (B.1.617.2) in India, and Omicron (B.1.1.529) in Botswana and South Africa (21–26). This has resulted in a catastrophic impact on global efforts against the SARS-CoV-2 pandemic, including vaccinations.

## Vaccine lessons learned from natural SARS-CoV-2 infection

Dysregulation of macrophages, neutrophils, Th17 cells, monocytes, basophils, eosinophils, megakaryocytes, and erythroid progenitor cells plays a role in severe COVID-19 disease (27–32). On the other hand, properly functioning CD4+, CD8+ cells, NK cells, and dendritic cells reduce the disease severity (33–37). For instance, in the lungs, tissue-resident memory CD8+ T cells are important for protection against repeated infection with respiratory viruses (33) and severe COVID-19 cases are also associated with CD8+ lymphopenia and Th17 polarization of T cells (34). Long-lasting B and T memory cells can persist in recovered individuals long after neutralizing antibody titers have waned and a good T follicular helper cell activation indicates a matured humoral immune response with specific memory B cells to rapidly respond to possible reinfection (38, 39). While the currently approved vaccines are based on a single spike protein epitope, the immunological memory after natural infection captures a diverse repertoire of SARS-CoV-2 epitopes for both B and T cell responses (40–42). Therefore, it is imperative to understand immune response to natural infection to better identify ‘correlates of protection’ against COVID-19. For instance, the nucleocapsid (N) protein is more conserved than S-protein in the coronavirus genus and one of the most abundant structural proteins in infected cells (43). N-protein levels were reported to regulate the severity of immune response to SARS-CoV-2 (44). Most of the recovered COVID-19 patients presented a stronger specific immune response against the SARS-CoV-2 N-protein and its fragments than the Receptor Binding Domain (RBD) of the S-protein (45). Patient sera during both the acute and the convalescent phase of COVID-19, showed IgM and IgG specific reactivity to N- and C-termini of the membrane (M) protein of SARS-CoV-2 (46). Antibodies against the M-protein show similar levels of reactivity as antibodies against the immunodominant epitopes of S or N proteins, but none of the vaccines approved in the US target the M-protein. Since emerging Variants of Concern (VOCs) show several mutations in the S- proteins, an in-depth study of M-protein as a vaccine target is required to move the evolutionary pressure away from S-protein. Using both S and N protein as vaccines have shown better virus neutralization at distal tissues sites (away from the primary respiratory site of infection) (47). With the emergence of the Omicron variant, the efficacy of single target (S-protein) mRNA vaccines has been wanning (48). The mRNA vaccine specifically targeting the omicron S-protein (mRNA-Omicron) did not perform better than the mRNA-1273 vaccine targeting the ancestral SARS-CoV-2 S-protein (49–53). These data suggest that targeting multiple SARS-CoV-2 proteins (S, N, M) would better replicate the natural COVID-19 infection and might trigger better protection against current and future SARS-CoV-2 variants.

## BBV152

**Figure 1:**
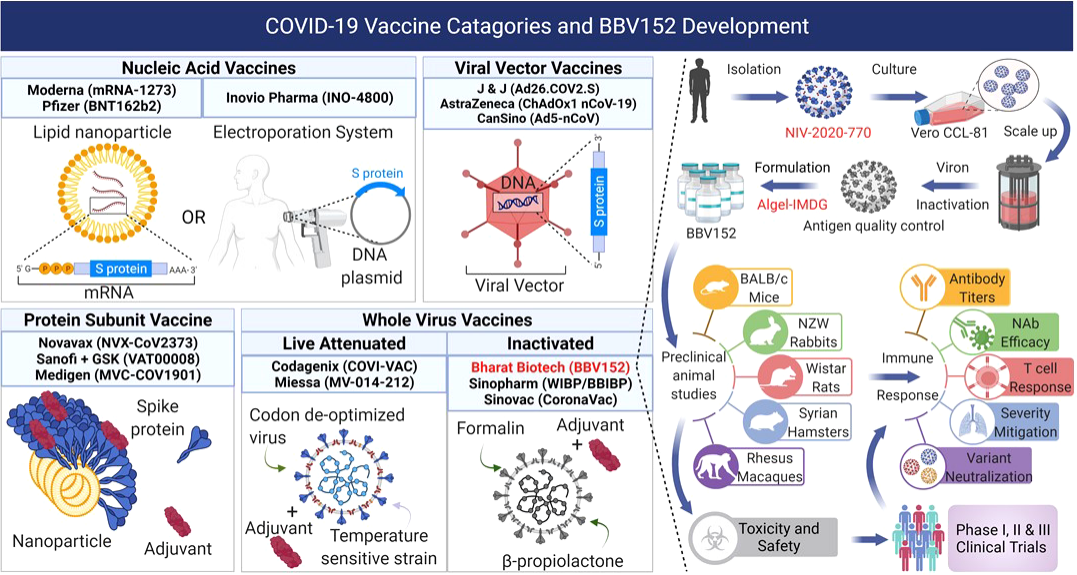
COVID-19 vaccines in development, clinical trials, or approved use, fall into four main categories: 1) Nucleic acid vaccines that use RNA or DNA to provide host cells with the instructions to make the viral antigens. 2) Viral vector vaccines use replicating or non-replicating, non-pathogenic viruses to deliver the genetic instructions to the host cells to produce antigens. 3) Protein subunit vaccines use fragments of antigens as proteins or assembled virus-like nanoparticles delivered directly into the host. 4) Whole virus vaccines use the complete virus that is either attenuated or completely inactivated. BBV152 is a whole virus inactivated vaccine produced from the NIV-2020-770 strain of SARS-CoV-2 strain by destroying the viral genetic material while leaving the antigens intact. The inactivated virus is formulated with the Algel-IMDG adjuvant (TLR7/8 agonist) and used for preclinical animal studies for safety and immunogenicity followed subsequently by Phase I, II, and III clinical trials to evaluate its safety and efficacy in human participants. Figure created with BioRender.com.

Multiple vaccines for SARS-CoV-2 are highly effective in reducing the COVID-19 pandemic burden. However, to meet the global need for billions of COVID-19 vaccine doses, a collective effort to identify, evaluate, validate, and manufacture effective vaccines is paramount. BBV152 is a whole-virion inactivated SARS-CoV-2 vaccine generated using the Asp614Gly (B.1.1.7) variant. BBV152 is the first vaccine formulated with a toll-like receptor 7/8 (TLR7/8) agonist molecule, imidazoquinoline gallamide (IMDG), adsorbed to alum and approved for use in a large population (54) (**Figure 1**). This vaccine is administered as two intramuscular 6µg doses, 4 weeks apart. In Phase I/II clinical trial, 98.3% of the candidates administered BBV152 developed antibodies against SARS-CoV-2 (seroconverted) (55). In a corresponding Phase III trial, BBV152 protected up to 77.8% of vaccinated candidates against symptomatic infection, 93.4% from severe disease, and 63.6% from asymptomatic disease (56).

## Preclinical development

Inactivated vaccines against viral diseases have well-established safety profiles and have been licensed and used for decades (57). The Vero cell vaccine manufacturing platform is well-characterized with proven safety in other licensed, live, and inactivated vaccines such as Ervebo (Ebola), Vepacel (Influenza), IMOVAX (Polio), VERORAB (Rabies), RotaTeq (Rotavirus) and ACAM2000 (Smallpox) (58–62). The BBV152 vaccine was developed from the SARS-CoV-2 strain (NIV-2020-770 - GISAID sequence EPI_ISL_420545 – G-clade) using the Vero CCL-81 system for sample propagation and virus isolation (**Figure 1**) (63–65). Inactivation of the β-propiolactone treated live virus was assessed by viral cytopathic activity and amplification while intact coronaviral morphology was verified by transmission electron microscopy, and the presence of intact S (S1, S2, and receptor binding (RBD) domains) and N-protein antigens was verified by Western blots and by immunoelectron microscopy (66).

**Figure 2:**
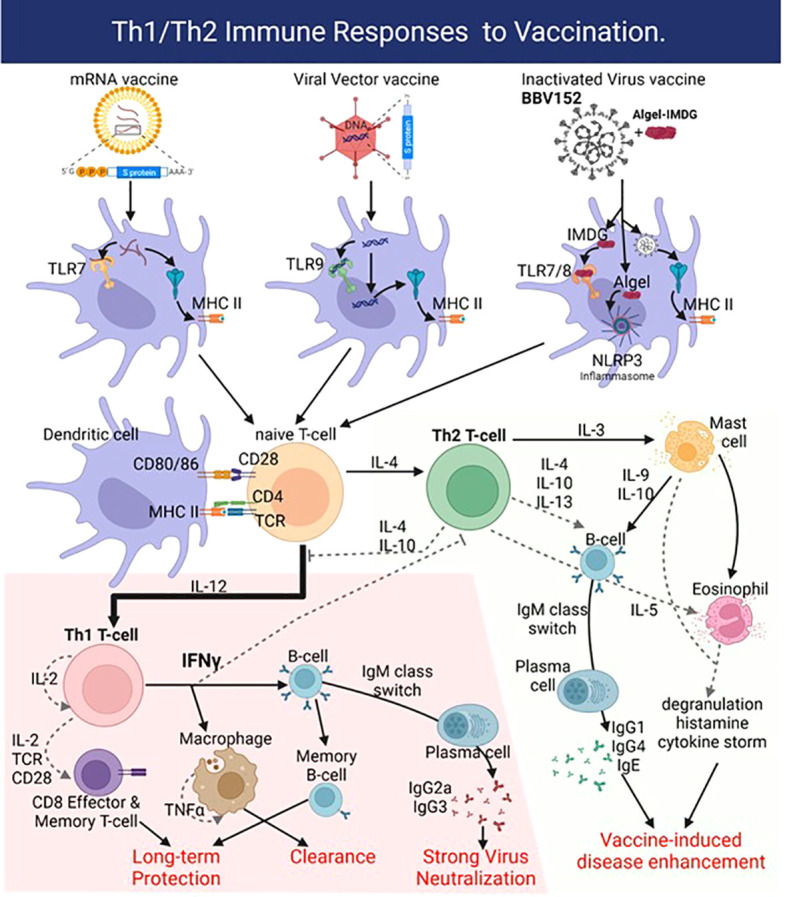
Vaccines deliver the SARS-CoV-2 antigen into dendritic cells (DC) using lipid nanoparticles (mRNA vaccines), adenoviral vectors, or in the case of BBV152 by using the inactivated SARS-CoV-2 virus. Adjuvant activity of the vaccines triggers innate sensors like the mRNA sensor Toll-like receptor 7 (TLR7) or double-strand DNA sensor TLR9 for mRNA and Viral Vector vaccines, respectively. The Algel-IMDG adjuvant in BBV152 activates TLR7/8 (IMDG) and the NLRP3 inflammasome (Algel). The resulting production of IL-12 drives naïve T-cells towards the pro-inflammatory Th1-biased response that generates strongly neutralizing antibodies (IgG1, IgG3 subtypes) and virus-specific CD8 effector T-cells to clear the infection. Generation of viral antigen-specific memory B- and CD8 T-cells affords long-term protection. Production of IL-4 however drives a Th2-biased response with high titers of IgG4 and IgE, along with recruitment and activation of mast cells and eosinophils, which causes vaccine-induced disease enhancement or antibody-dependent enhancement. Figure created with BioRender.com.

Vaccine-induced disease enhancement due to Th2-like immunity is a concern for patients vaccinated against SARS-CoV-2 (**Figure 2**) (67). Several studies show that patients with higher antibody titers against SARS-CoV-2 were associated with higher antigen levels (viral loads) and more prolonged exposure leading to more severe disease symptoms (14, 19, 68). Patients with mild and asymptomatic COVID-19 infections show high levels of anti-SARS-CoV-2 T-cell (Th1) responses (69, 70). These data suggest that strong Th1 responses correlate with mild clinical presentations, whereas strong Th2 responses correlate with severe COVID-19. BBV152 circumvents this Th2 bias using a new IMDG (TLR7/8 agonist) class adjuvant that induces strong type I interferon responses from antigen-presenting cells such as dendritic cells and monocytes-macrophages leading to the development of a Th1 cell-based immunity instead of a pathogenic Th2 humoral immunity (**Figure 2**) (71). Like the mRNA COVID-19 vaccines, BBV152 also induces a strong CD8 T cell response in vaccinated individuals (72, 73). Preclinical BBV152 immunogenicity and safety studies done on multiple animal models are summarized in **Table 1**. The maximum tolerated dose of TLR7/8 agonist (IMDG) in Algel-IMDG formulation was 30 ug/animal in mice and rats (76). The BBV152-Algel-IMDG formulations were evaluated as safe in mouse, rat, and rabbit models (**Figure 1**) by repeated-dose toxicity, mutagenicity, histopathology, and by clinical pathology investigations such as hematology, clinical biochemistry, coagulation parameters, and urinalysis monitoring. Necropsy with organ histopathology revealed no significant difference between animals treated with antigen alone, adjuvants alone, or adjuvanted vaccine. Animals administered adjuvanted formulations showed local inflammatory changes characterized by mild infiltration of mononuclear cells and the presence of macrophages at the injection site (76). BBV152 immunogenicity was tested in BALB/c mice, Wistar rats, and in New Zealand white rabbits on days 0, 7, and 14. The respective sera were evaluated for antibody binding by ELISA and efficacy of live SARS-CoV-2 neutralization by plaque reduction assays. Animals administered BBV152 (adjuvanted vaccine) showed higher antibody binding (to S1, RBD, and N proteins) and higher neutralizing antibody (NAb) titers than those administered the antigen alone (76). High IgG2a/IgG1 in day 21 serum samples, higher levels of interferon-γ (IFNγ), IL-2, 4, 6, 17A, TNFα, and higher populations of CD4 T_fh_-cells (follicular helper) suggest that Algel-IMDG (TLR7/8 agonist) adjuvant induces Th1 biased, T-cell mediated, protective immunity (66, 76).

**Table 1:** BBV152 preclinical immunogenicity and safety studies.

Study type	Model	Vaccination schedule	Vaccination route	Infection dose	Readouts					Publication
					Virus levels	NAb titers	Binding levels	Histopathology	Safety	
Immunogenicity	BALB/c mice	day 0 & 7 - 0.15 to 0.3µg antigen + Algel-IMDG	Intraperitoneal	-	-	High efficacy - MNT_50_ - day 7 PV	High S1, RBD, & N specific Ab titers- day 7 PV	-	-	Ganneru et al., 2020 (66)
			Intramuscular							
Long term Immunogenicity			Intramuscular			MNT_50_ - day 98 PV	S1-specific Ab titers- day 98 PV			
Repeated dose toxicity	BALB/c mice	day 0, 7 & 14 - 0.15 to 0.3 µg antigen + 6 µg Algel-IMDG	Intraperitoneal	-	-	-	-	Mild WBC infiltration at the injection site with no skin reactions. All organs show normal morphology.	Body temperature, leucocyte counts, clinical biochemistry parameters and urine analysis reports within normal range.	
	Wistar rats	day 0, 7 & 14 - 3 to 6 µg antigen + 30 µg Algel-IMDG	Intramuscular							
	New Zealand Rabbits									
Maximum tolerated dose	Swiss Albino mice	day 0, 7 & 14 - 9 µg antigen + 30 µg Algel-IMDG								
	Wistar rats									
Viral challenge	Syrian Hamsters	day 0, 14 & 35 - 3 to 6 µg antigen + 6 µg Algel-IMDG	Intramuscular	Intranasal 0.1 ml - 10^5.5 TCID_50_ SARS-CoV-2 on day 50 PV	low viral titers and RNA from throat swabs and nasal washes on days 3, 7 & 15 PI	-	-	-	-	Mohandas et al., 2021 (74)
Immunogenicity					-	Improved NAb PRNT_50_) against NIV-2020-770 strain days 12, 21, 48 PV	Elevated anti-spike IgG & IgG2 levels on days 3, 7 and 14 PI	-	-	
Protection Study					-	-	-	Vaccinated animal lungs and trachea protected from pneumonia	Vaccinated animals show higher IFNg, IL-12 and lower IL-4 and IL-6 levels PI.	
Viral challenge	Rhesus Macaques	day 0 & 14 - 3 to 6 µg antigen + 6 µg Algel-IMDG	Intramuscular	1ml Intratracheal and 0.25 ml in each nostril - 10^6.5 TCID_50_ SARS-CoV-2 on day 28 PV	low viral titers and RNA from throat swabs and nasal washes on days 1, 3, 5 & 7 PI	-	-	-	-	Yadav et al., 2021 (75)
Immunogenicity					-	Improved NAb PRNT_50_) against the vaccine strain & 2 heterologous strains on days 0, 1, 3, 5 & 7 PV	Elevated anti-spike, RBD and N-protein IgG and IgG2 levels on days 0 & 7 PI	-	-	
Protection Study					-	-	-	Vaccinated animal lungs and trachea protected from pneumonia	No viral RNA was detected from pulmonary or extra-pulmonary tissues of vaccinated animals.	

## BBV152 immunogenicity and protection in Syrian hamsters and rhesus macaques

Among various small laboratory animal models, Syrian hamsters were found to be a suitable model for SARS-CoV-2 research, as the virus has been shown to replicate both in the upper and lower respiratory tract (77) (**Table 1**). Syrian hamsters (n=9/group) immunized with phosphate-buffered saline (PBS-control), BBV152-Algel or BBV152-Algel-IMDG (3 µg or 6 µg) on days 0, 14, and 35 and inoculated on day 50 with intranasal SARS-CoV-2 (NIV-2020-770) median tissue culture infectious dose (TCID_50_) of 10^5.5^ (74). By 21 days post-immunization, hamsters receiving BBV152 with Algel-IMDG adjuvant produced significantly higher NAbs than the Algel alone group.

BBV152 vaccinated hamsters showed rapid clearance of SARS-CoV-2 from lungs, trachea, nasal turbinates, and extra-pulmonary tissues while also showing lower inflammation mediated alveolar damage, hemorrhages, inflammatory cell infiltration, hyaline membrane formation, and eosinophilic edematous exudate than the PBS-treated control group (**Figure 1**) (74). A similar assessment of immunogenicity of the three BBV152 formulations in rhesus macaques (75) (**Table 1**) showed protective efficacy against SARS-CoV-2 pneumonia and preserved the normal histology of the lungs. BBV152-treated macaques showed normal post-infection oxygen saturation (SpO_2_), lower levels of pro-inflammatory cytokine IL-6, and protection against post-infection CD8 T-cell lymphopenia. The lung histopathology of BBV152-treated animals showed no signs of eosinophilic infiltration, indicating no vaccine-enhanced disease (**Figure 1**) (75).

## BBV152 safety phase I trial

BBV152 safety and immunogenicity in humans were first evaluated in a double-blind, multicenter, randomized, controlled Phase I trial (ClinicalTrials.gov: NCT04471519) (**Table 2**). Individuals with positive SARS-CoV-2 nucleic acid and/or serology tests were excluded. Among the 375 healthy adults aged 18 to 55 years at 11 hospitals across India, three groups of 100 were randomly assigned to receive either one of three BBV152 formulations (3 or 6 µg with Algel-IMDG or 6 µg with Algel), and 75 were assigned to an Algel only control vaccine group. Intramuscular vaccine/control doses were administered on the day of randomization and again on day 14. As the primary outcome, solicited local and systemic adverse reactions were reported by 17 (17%; 95% CI, 10.5–26.1) participants in the 3 µg BBV152-Algel-IMDG group, 21 (21%; 13.8–30.5) in the 6 µg BBV152- Algel-IMDG group, 14 (14%; 95% CI, 8.1–22.7) in the 6 µg BBV152-Algel group, and 10 (10%; 95% CI, 6.9–23.6) in the Algel-only group. The most common solicited adverse events were injection site pain (17 [5%] of 375 participants), headache (13 [3%]), fatigue (11 [3%]), fever (nine [2%]), and nausea or vomiting (seven [2%]) (**Table 3**). All solicited adverse events were mild (43 [69%] of 62) or moderate (19 [31%]) and were more frequent after the first dose. BBV152 immunogenicity was measured as the study’s secondary outcome using both IgG levels (Anti-S1, Anti-RBD, and Anti-N IgG1) and appearance of NAbs from baseline to days 14, 28, 42, 104, and 194. Seroconversion rates were 87.9, 91.9, and 82.8% in the 3 µg with Algel-IMDG, 6 µg with Algel-IMDG, and 6 µg with Algel groups, respectively. Analysis performed on randomly selected blood samples at one study site, showed enhanced CD3, CD4, and CD8 T-cell (Th1) activation measured by IFN-γ ELISpot responses against SARS-CoV-2 in both Algel-IMDG vaccinated groups on day 28 (54) (**Table 2**).

**Table 2:** BBV152 Phase I, II and III immunogenicity.

Clinical Trials	Participants	Immune Readout												
Phase I - NCT04471519 (Ella et al., 2021 PMCID: PMC8221739)	N = 375	Post Vaccination Seroconversion % (95% CI)			Post Vaccination NAb MNT50 GMT (95% CI)			Cell-mediated response GMT (95% CI)			ELISPOT (IFNg)	Post vaccination ELISA Titer GMT (95% CI)		
		S1-protein - day28	RBD-protein - day28	N-protein - day28	MNT50 - Day 0	MNT50 - Day 14	MNT50 - Day 28	% CD3	% CD4	% CD8	Day 28 Median (IQR)	S1-protein - day28	RBD-protein - day28	N-protein - day28
	N=100 - 3 µg antigen + 6 µg Algel-IMDG	93.8% (87.7 - 97.5)	83.2% (74.9 - 89.6)	89.9% (82.2 - 94.4)	6.21 (5.9 - 6.5)	9·14 (8·1 - 10·4)	61·70 (49·5 - 76·9)	0.5 (0.3 - 1.2)	0.4 (0.2 - 1.0)	0.04 (0.01 - 0.2)	105 (8.5 - 166.0)	4955.7 (4192.9 - 5857.3)	2622.4 (2307.7 - 2980.1)	3148.2 (2692.5 - 3681.1)
	N=100 - 6 µg antigen + 6 µg Algel-IMDG	93.3% (86.6 - 97.3)	91.4% (84.2 - 95.9)	85.6% (77.5 - 91.2)	6·01 (5·8 - 6·2)	11·20 (9·6 - 13·0)	66·4 (53·4 - 82·4)	0.7 (0.4 - 2.1)	0.8 (0.3 - 1.0)	0.05 (0.01 - 1.3)	55.0 (22.0 - 173.8)	5771.1 (4793.6 - 6948.0)	3138.3 (2747.8 - 3584.4)	4112.5 (3410.6 - 4958.8)
	N=100 - 6 µg antigen + 6 µg Algel	97.9% (92.6 - 99.7)	94.7% (88.1 - 98.3)	89.5% (81.5 - 94.8)	5·95 (5·8 - 6·1)	9·45 (8·2 - 10·9)	48·00 (37·7 - 61·1)	0.2 (0.1 - 0.5)	0.1 (0.1 - 0.3)	0.02 (0.01 - 0.05)	31.5 (16.0 - 121.0)	6286.1 (5339.4 - 7400.8)	3681.9 (3174.6 - 4270.2)	2981.6 (2546.2 - 3490.1)
	N=75 - 6 µg Algel	65.8% (54.3 - 76.1)	55.7% (44.1 - 66.9)	49.4% (37.9 - 60.9)	6·13 (5·8 - 6·4)	6·07 (5·9 - 6·3)	7·20 (6·4 - 8·1)	0.07 (0.04 - 0.6)	0.08 (0.02 - 0.3)	0.01 (0.01 - 0.04)	3.0 (1.0 - 23.0)	2000.0 (1654.6 - 2417.5)	1621.5 (1364.4 - 1927.1)	1651.4 (1375.5 - 1982.6)
**Phase II - NCT04471519 (Ella et al., 2021 PMCID: PMC7825810)**	**N = 380**	**Post Vaccination Seroconversion % (95% CI)**			**Post Vaccination NAb GMT (95% CI)**			**Cell-mediated response GMT on day 42 (95% CI)**		**Post vaccination ELISA Titer GMT (95% CI)**						
		S1-protein - day28	RBD-protein - day28	N-protein - day28	MNT50 - Day 28	MNT50 - Day 42	MNT50 - Day 56	IL-2 (pg/ml)	IL-5 (pg/ml)	S1-protein - day28		RBD-protein - day28		N-protein - day28
	N=190 - 3 µg antigen + 6 µg Algel-IMDG	71·2% (64·1–77·6)	58·7% (51·2–65·9)	72·3% (65·2–78·6)	12.6 (10.8 - 14.7)	78.5 (64.6 - 95.2)	92.5 (77.7 - 110.2)	42.13 (31.0 - 53.2)	33.4 (29.2 - 37.5)	2574·2 (2228·9–2973·1)		1962·7 (1726·2–2231·6)		2734·1 (2375·1–3147·5)
	N=190 - 6 µg antigen + 6 µg Algel-IMDG	65·0% (57·5–72·0)	58·2% (50·6–65·6)	71·2% (63·9–77·7)	12.0 (10.2 - 14.0)	134.8 (144.4 - 158.8)	160.1 (135.8 - 188.8)	28.1 (22.9 - 33.3)	30.9 (27.2 - 34.6)	2240·5 (1942·4–2584·5)		2031·6 (1777·3–2322·3)		2490·4 (2161·7–2869·2)
		S1-protein - day42	RBD-protein - day42	N-protein - day42	PRNT50 - Day 28	PRNT50 - Day 42	PRNT50 - Day 56	IFNg (pg/ml)	IL-13 (pg/ml)	S1-protein - day42		RBD-protein - day42		N-protein - day42
	N=190 - 3 µg antigen + 6 µg Algel-IMDG	98·4% (95·3–99·7)	94·0% (89·6, 97·0)	97·3% (93·8–99·1)	1.23 (0.78 - 1.94)	78.4 (54.8 - 112.0)	100.9 (74.1 - 137.4)	1167.2 (445.9 - 1888)	20.1 (14.6 - 25.6)	11528·8 (10 002·7–13 287·8)		5572·3 (4897·5, 6339·9)		8957·2 (7778·6–10314·3)
	N=190 - 6 µg antigen + 6 µg Algel-IMDG	98·3% (95·1–99·7)	93·2% (88·5, 96·5)	95·5% (91·3–98·0)	1.54 (0.99 - 2.4)	161.8 (126.2 - 207.4)	197.0 (155.6 - 249.4)	1082.5 (110.9 - 2054)	16.3 (9.4 - 23.1)	10040·0 (8667·0–11 630·5)		4980·8 (4366·7, 5681·3)		9211·2 (7939·3–10 686·8)
		S1-protein - day56	RBD-protein - day56	N-protein - day56				Th1:Th2 (IFNg + IL-2 + TNFa / IL-5+IL-13)		S1-protein - day56		RBD-protein - day56		N-protein - day56
	N=190 - 3 µg antigen + 6 µg Algel-IMDG	98·4% (95·3–99·7)	96·2% (92·3, 98·5)	97·3% (95·3–100·0)				59.2 (48.5 - 69.7)		10413·9 (9142·4–11 862·2)		5874·0 (5194·8, 6642·0)		8626·0 (7528·6–9883·4)
	N=190 - 6 µg antigen + 6 µg Algel-IMDG	96·6% (92·8–98·8)	94·4% (89·9, 97·3)	96·6% (92·8–98·8)				42.5 (28.6 - 56.3)		9541·6 (8245·9–11 041·0)		5558·0 (4859·9, 6356·5)		8754·0 (7589·4–10 097·4)
**Phase III - NCT04641481 (Ella et al., 2021 PMCID: PMC8584828)**	**N=25798**	**BBV152 Vaccine Efficacy**					**Post Vaccination (Day 56) NAb MNT50 GMT (95% CI)**			**Post vaccination ELISA Titer GMT (95% CI)**				
	Symptomatic COVID	Severe COVID	Asymptomatic COVID	Symptomatic < 60 yrs	Symptomatic ≥ 60 yrs	Age	≥18–<60 years	≥ 60 years		S1-protein - day56	RBD-protein - day56	N-protein - day56		
	Total cases n/N (%)	130/16 973 (0·8%)	16/16 973 (0·1%)	46/6289 (0·7%)	109/15 115 (0·7%)	21/1858 (1·1%)	BBV152 (N=386)	129.9 (114.3 - 147.6)	101.2 (70.0 - 146.3)	BBV152 (N=397)	9742 (8949–10 606)	4124 (3731–4557)	4161 (3736–4633)	
	BBV152 n/N (%)	24/8471 (0·3%)	1/8471 (<0·1%)	13/3248 (0·4%)	19/7578 (0·3%)	5/893 (0·6%)	Placebo (N=119)	12.9 (10.1 - 16.5)	19.1 (9.0 - 40.5)	Placebo (N=125)	1528 (1323–1765)	1443 (1261–1651)	1485 (1275–1730)	
	Placebo n/N (%)	106/8502 (1·2%)	15/8502 (0·2%)	33/3041 (1·1%)	90/7537 (1·2%)	16/965 (1·7%)	Gender	Male	Female					
	Vaccine efficacy, % (95% CI)	77·8% (65·2–86·4)	93·4% (57·1–99·8)	63·6% (29·0–82·4)	79·4% (66·0–88·2)	67·8% (8·0–90·0)	BBV152 (N=386)	118.2 (101.0 - 138.3)	138.4 (114.4 - 167.3)					
		**BBV152 Vaccine Efficacy against SARS-CoV-2 variants**					Placebo (N=119)	14.1 (10.4 - 19.2)	12.9 (8.8 - 19.0)					
		All Variants	Delta (B.1.617.2)	Kappa (B.1.617.1)	Alpha (B1.1.7)	Other	Baseline SARS-CoV-2	Positive	Negative					
	Total cases n/N (%)	79 (0·5%)	50 (0·3%)	11 (0·1%)	4 (<0·1%)	14 (0·1%)	BBV152 (N=386)	194.3 (134.4 - 280.9)	118.0 (104.0 - 134.0)					
	BBV152 n/N (%)	18 (0·2%)	13 (0·2%)	1 (<0·1%)	1 (<0·1%)	3 (<0·1%)	Placebo (N=119)	27.4 (14.0 - 53.5)	11.9 (9.3 - 15.2)					
	Placebo n/N (%)	61 (0·7%)	37 (0·4%)	10 (0·1%)	3 (<0·1%)	11 (0·1%)								
	Vaccine efficacy, % (95% CI)	70·8% (50·0 to 83·8)	65·2% (33·1 to 83·0)	90·1% (30·4 to 99·8)	–	73·0% (−2·2 to 95·2)								

Table 3BBV152 Phase I, II and III adverse events.

**Table:** 

Clinical Trials	Participants	Adverse events																												
**Phase I - NCT04471519 (Ella et al., 2021 PMCID: PMC8221739)**	**N = 375**	**Local N (%; 95% CI)**								**Systemic N (%; 95% CI)**																				**Total N (%; 95% CI)**
		Pain at injection site				Swelling				Fever				Bodyache				Fatigue				Headache				Nausea				
		Mild		Moderate		Mild		Moderate		Mild		Moderate		Mild		Moderate		Mild		Moderate		Mild		Moderate		Mild		Moderate		
		Dose 1	Dose 2	Dose 1	Dose 2	Dose 1	Dose 2	Dose 1	Dose 2	Dose 1	Dose 2	Dose 1	Dose 2	Dose 1	Dose 2	Dose 1	Dose 2	Dose 1	Dose 2	Dose 1	Dose 2	Dose 1	Dose 2	Dose 1	Dose 2	Dose 1	Dose 2	Dose 1	Dose 2	
	N=100 - 3 µg antigen + 6 µg Algel-IMDG	4 (4%; 1.1- 9·9)	2 (2%; 0·2–7·0)	1 (1%; 0·0–5·5)	0	0	0	0	0	0	2 (2%; 0·2–7·0)	0	0	0	0	0	1 (1%; 0·0–5·5)	1 (1%; 0·0–5·4)	1 (1%; 0·0–5·4)	2 (2%; 0·2–7·0)	1 (1%; 0·0–5·5)	1 (1%; 0·0–5·5)	0	0	0	1 (1%; 0·0–5·5)	0	0	0	**17 (17%; 10·5–26·1)**
	N=100 - 6 µg antigen + 6 µg Algel-IMDG	4 (4%; 1·1–9·9)	1 (1%; 0·03–5·5)	1 (1%; 0·0–5·5)	0	0	0	0	0	1 (1%; 0·0–5·5)	1 (1%; 0·0–5·5)	1 (1%; 0·0–5·5)	0	1 (1%; 0·03–5·5)	0	1 (1%; 0·0–5·5)	0	0	0	3 (3%; 0·6–8·5)	0	2 (2%; 0·2–7·0)	0	3 (3%; 0·6–8·5)	0	2 (2%; 0·2–7·0)	0	0	0	**21 (21%; 13·8–30·5)**
	N=100 - 6 µg antigen + 6 µg Algel	1 (1%; 0·0–5·5)	1 (1%; 0·0–5·5)	0	0	0	0	0	0	1 (1%; 0·0–5·5)	1 (1%; 0·0–5·5)	2 (2%; 0·2–7·0)	0	0	0	1 (1%; 0·0–5·5)	0	0	3 (3%; 0·6–8·5)	0	0	0	0	2 (2%; 0·2–7·0)	0	2 (2%; 0·2–7·0)	0	0	0	**21 (21%; 13·8–30·5)**
	N=75 - 6 µg Algel	2 (3%; 0·3–9·3)	0	0	0	1 (1%; 0·0–7·2)	0	0	0	0	0	0	0	0	0	0	0	0	0	0	0	5 (7%; 2·2–15)	0	0	0	2 (3%; 0·3–9·3)	0	0	0	**10 (10%; 6·9–23·6)**

**Table:** 

Clinical Trials	Participants		Adverse events																							
**Phase II - NCT04471519 (Ella et al., 2021 PMCID: PMC7825810)**	**N = 380**		**Local n(%)**										**Total Local N (%; 95% CI)**	**Systemic n(%)**												**Total Systemic N (%; 95% CI)**
			Pain at injection site		Redness at injection site		Itching		Stiffness		Weakness in injection arm			Fever		Bodyache		Fatigue		Headache		Weakness		Rashes		
			Mild	Moderate	Mild	Moderate	Mild	Moderate	Mild	Moderate	Mild	Moderate		Mild	Moderate	Mild	Moderate	Mild	Moderate	Mild	Moderate	Mild	Moderate	Mild	Moderate	
	Dose 1	N=190 - 3 µg antigen + 6 µg Algel-IMDG	5 (3%)	1 (1%)	1 (1%)	0	1 (1%)	0	1 (1%)	0	0	0	**9 (4·7%; 2·2–8·8)**	2 (1%)	1 (1%)	0	0	4 (2%)	0	2 (1%)	0	0	0	0	0	**9 (4·7%; 2·2–8·8)**
		N=190 - 6 µg antigen + 6 µg Algel-IMDG	6 (3%)	0	1 (1%)	0	1 (1%)	0	0	0	0	0	**8 (4·2%; 1·8–8·1)**	5 (3%)	3 (2%)	2 (1%)	1 (1%)	1 (1%)	0	1 (1%)	0	0	1 (1%)	0	0	**14 (7·4%; 4·1–12·1)**
	Dose 2	N=190 - 3 µg antigen + 6 µg Algel-IMDG	7 (4%)	0	0	0	0	0	0	0	1 (1%)	0	**8 (4·2%; 1·8–8·1)**	5 (3%)	0	1 (1%)	0	3 (2%)	0	1 (1%)	0	1 (1%)	0	1 (1%)	–	**7 (3·7%; 1·6–7·7)**
		N=190 - 6 µg antigen + 6 µg Algel-IMDG	4 (2%)	1 (1%)	0	0	2 (1%)	0	0	0	0	0	**12 (6·3%; 3·3–10·8)**	4 (2%)	0	2 (1%)	0	0	0	2 (1%)	1 (1%)	2 (1%)	0	0	0	**11 (5·8%; 3·0–10·1)**

**Table:** 

**Phase III - NCT04641481 (Ella et al., 2021 PMCID: PMC8584828)**	**N = 25753**		**Local n (%)**							**Systemic n (%)**								**Total n (%)**
			Pain	Redness	Induration	Swelling	Mild	Moderate	Severe	Fever	Fatigue	Chills	Headache	Myalgia	Arthralgia	Nausea	Vomiting	
	BBV152 (N = 12,879)	Dose 1	392 (3·04)	33 (0·26)	32 (0·25)	21 (0·16)	421 (3·27)	10 (0·08)	0	108 (0·84)	52 (0·40)	28 (0·22)	128 (0·99)	49 (0·38)	17 (0·13)	17 (0·13)	12 (0·09)	**1597 (12·4)**
		Dose 2	233 (1·81)	21 (0·16)	18 (0·14)	14 (0·11)	272 (2·11)	6 (0·05)	0	86 (0·67)	41 (0·32)	9 (0·07)	86 (0·67)	37 (0·29)	12 (0·09)	14 (0·11)	6 (0·05)	
	Placebo (N = 12,874)	Dose 1	358 (2·78)	26 (0·20)	26 (0·20)	32 (0·25)	392 (3·05)	7 (0·05)	0	81 (0·63)	41 (0·32)	22 (0·17)	111 (0·86)	28 (0·22)	17 (0·13)	12 (0·09)	8 (0·06)	**1597 (12·4)**
		Dose 2	208 (1·62)	25 (0·19)	18 (0·14)	16 (0·12)	254 (1·97)	6 (0·05)	0	79 (0·61)	20 (0·16)	16 (0·12)	70 (0·54)	28 (0·22)	17 (0·13)	10 (0·08)	8 (0·06)	

## BBV152 immunogenicity phase II trial

Following the tolerable safety outcomes and enhanced immune responses for BBV152 reported in the Phase I trial, both Algel-IMDG formulations were selected for a double-blind, randomized, multicenter Phase II trial in healthy adults and adolescents (aged 12 to 65 years) at 9 hospitals in India. After excluding participants already positive for SARS-CoV-2 (by serology or RT-PCR), 380 candidates were randomly assigned (1:1) to receive either 3 µg or 6 µg BBV152 with Algel-IMDG. As the primary study outcome, the group receiving 6 µg BBV152 with Algel-IMDG showed significantly higher titers of anti-SARS-CoV-2 NAbs and 98.3% [95% CI, 95.1–99.6] seroconversion rates (defined as a post-vaccination titer that was at least four-fold higher than the baseline) (55). IgG antibodies to all epitopes (S1 glycoprotein, RBD, and N protein) were detected (**Table 2**). Secondary outcomes revealed a Th1-biased response induced in both groups, measured by cytokine response (high Th1 cytokines - IFNγ, IL-2, and TNFα and low Th2 cytokines - IL-5, IL-10, and IL-13) at 2 weeks after the second vaccine dose (day 42). Additionally, BBV152 induced CD4+ CD45RO+ memory T-cell responses with elevated co-stimulatory marker CD27 indicating the activation of the antigen recall memory T-cell response against SARS-CoV-2 (55). Candidates with an existing co-infection were excluded to avoid confounding the vaccine generated immune responses with Th2/Th17 immune responses to other pathogens. No serious adverse events were reported in the study. Most adverse events were mild and resolved within 24 hours of onset. No association between the dose of vaccine (3 µg or 6 µg BBV152) and the number of adverse events was observed. The most common solicited adverse event was injection site pain (**Table 3**).

## BBV152 efficacy phase III trial

In the Phase II trial, BBV152 showed better immunogenicity, safety, and enhanced Th1 biased anti-SARS-CoV-2 immune responses compared to the Phase I trial. Therefore the 6 µg BBV152 with Algel-IMDG formulation was selected for the double-blind, randomized, controlled Phase III efficacy trial (Clinicaltrials.gov: NCT04641481). This study recruited 25798 participants between November 16, 2020, and January 7, 2021, of which 24419 were randomized to receive two doses of BBV152 (n = 12,221) or placebo (n = 12,198). The primary outcome was to assess the efficacy of BBV152 in preventing symptomatic COVID-19 (confirmed by RT-PCR) in a case-driven manner, along with sub-group analyses of asymptomatic and symptomatic efficacy. In a follow-up, at least two weeks after the second vaccination, 130 (0.77%) cases of symptomatic COVID-19 were reported among 16,973 participants; 24 in the BBV152 group and 106 in the placebo group, bringing the overall vaccine efficacy to 77.8% (95% CI: 65.2–86.4) (**Table 2**). One candidate from the vaccine group and 15 from the placebo group developed severe COVID-19 symptoms, giving BBV152 a protective efficacy of 93.4% against severe disease, while the efficacy against asymptomatic COVID-19 was 63.6% (95% CI, 29.0–82.4) (56). BBV152 protection in elderly (>60 yrs.) candidates was 67.8% (95% CI, 8.0–90.0) and in participants younger than 60 years was 79.4% (95% CI, 66.0–88.2). BBV152 was well tolerated with no differences in the distributions of solicited, unsolicited, or serious adverse events between vaccine and placebo groups. No cases of anaphylaxis or vaccine-related deaths were reported (**Table 3**).

## BBV152 against variants of concern

**Figure 3:**
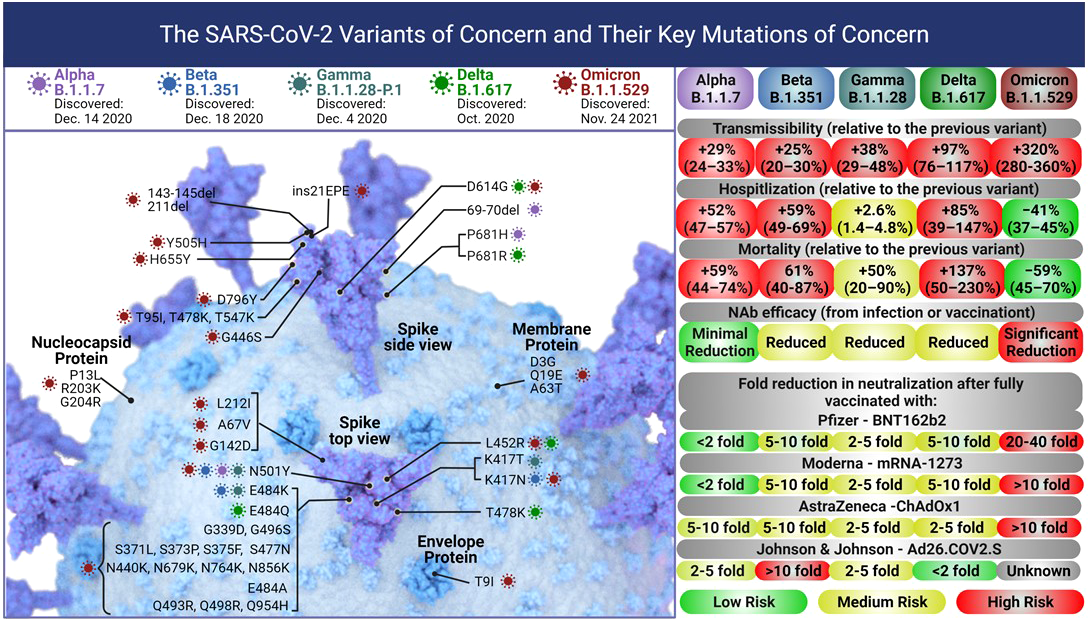
Each of the five SARS – CoV-2 variants of concern accumulated mutations in their Spike protein leading to changes in their transmissibility, and severity of infections leading to hospitalization or death. The current globally dominant variant, Omicron (B.1.1.529) has more than 50 mutations including 32 on the Spike protein and 15 on the RBD alone. These mutations have significantly increased the transmissibility of Omicron over the Delta variant. The severity (hospitalization and death) due to the Omicron variant is lower than Delta, however, the vaccine efficacy against Omicron drops significantly. Figure created with BioRender.com.

The rapid surge of SARS-CoV-2 cases due to the five variants of concern has resulted in a catastrophic impact on global efforts against the SARS-CoV-2 pandemic including vaccinations. Alpha (B.1.1.7), Beta (B.1.351), Gamma (B.1.1.28-P.1), Delta (B.1.617.2), and Omicron (B.1.1.529) variants pose serious public health concerns due to higher transmissibility, immune escape, and disease severity (**Figure 3**). The root of such concerns is due to the structure of these variants compared with the other known SARS-CoV-2 lineages. The Alpha (B.1.1.7) variant carries 8 mutations in spike-RBD, specifically N501Y, which enhances viral attachment to angiotensin-converting enzyme 2 (ACE2) on human cells (78). The Beta (B.1.351) variant has additional E484K and K417N mutations on the RBD, the Gamma (B.1.1.28) variant has a new L452R spike mutation plus two new mutations A119S and M234I in the N-protein, while the D614G, T478K, and P681R mutations were of concern in the Delta (B.1.617.2) variant (21, 23, 79, 80). The heavily mutated Omicron (B.1.1.529) variant spike shows 30 amino acid changes, three deletions, one insertion in the spike-RBD protein, and 3 mutations at the furin cleavage site that increase its infectivity (**Figure 3**) (81–84). Recent studies suggest that since most of the vaccine candidates are either recombinant or specifically target a single epitope of the original spike sequence, their immune response might not be as efficient against the new variants (85–89). An open-label, clinical intervention trial study done on the 4^th^ dose of mRNA vaccines (Pfizer - BNT162b2 or Moderna - mRNA1273) reported the vaccine efficacy against Omicron infection at 30% (95%CI, -9% to 55%) and 11% (95%CI, -43% to +43%) for BNT162b2 and mRNA1273, respectively while local and systemic adverse reactions were reported in 80% and 40% recipients, respectively (48).

Sera from selected BBV152 vaccinated candidates of Phase I/II clinical trials (ClinicalTrials.gov: NCT04471519) were tested against the five VOC and the vaccine strain D614G, using plaque reduction neutralization test (PRNT_50_) assays, with the seroconversion rates of neutralizing antibodies being 98.6% (90). The sera from BBV152-vaccinated individuals could neutralize Alpha (B.1.1.7) (PRNT_50_-GMT: D614G = 700, Alpha = 670) (91) and Gamma (B.1.1.28.2) variants (PRNT_50_-GMT: D614G = 337.5, Gamma= 175.7) (92) with similar efficacy as D614G. For Beta (B.1.351) neutralization efficacy dropped 3-fold compared to D614 (PRNT_50_-GMT: D614G = 187.5, Beta= 61.57) (93). Delta (B.1.617.2) and Omicron (B.1.1.529) variants were neutralized at 1.5and 9.4-fold lower efficacy than D614G (PRNT_50_-GMT: D614G=706, Delta=480, Omicron=75) (94). All BBV152 boosted candidates showed neutralizing activity against the Delta variant, while over 90% showed neutralizing activity against the Omicron variant (94). Similar studies using higher drop in neutralization efficacy was observed with mRNA vaccines like BNT162b2 (Pfizer) (6.5-fold) and mRNA-1273 (Moderna) (8.6-fold) while the viral vector vaccine ChAdOx1 nCoV-19 (Astra Zeneca) showed an 86-fold reduction in efficacy against Beta (B.1.351) variant (21, 95, 96). Similarly, reduced Delta (B.1.617.2) variant neutralization was observed with the sera of BNT162b2 mRNA and single-dose ChAdOx1 nCoV-19 vaccinated individuals (79).

## Protection with BBV152 homologous/heterologous prime-boost vaccination

BBV152 Phase I/II clinical trials reported an acceptable safety profile and enhanced Th1 (IFNγ producing CD4+ T-cells) and humoral responses (54, 55, 90). Based on the interim Phase III data, BBV152 was approved for use in India with over 313 million vaccine doses already administered by April 18^th^, 2022 (https://dashboard.cowin.gov.in/). Long-term immune responses can be enhanced by inducing trained immunity and down-regulating innate immune tolerance (97–99). To dissect the systemic immune responses induced by BBV152, the plasma levels of a panel of cytokines and chemokines were measured in 44 prime-boost vaccine recipients (100). While the first dose of the vaccine does not induce any significant changes in plasma cytokine levels, the second BBV152 dose enhanced plasma levels of Th1 (IFNγ, IL-2, TNFα), and Th2 (IL-4, IL-5, IL-10, and IL-13) cytokines as well as IL-17A and other pro-inflammatory cytokines (**Figure 2**). This was accompanied by elevated chemokines CCL4, CXCL1, CXCL2, and CX3CL1. A strong Th1 response correlates with milder COVID-19 symptoms (14, 19, 68). Although this study does not measure vaccine or ligand-specific immune responses, the induction of systemic cytokines indicates a robust systemic immune response following the BBV152 booster dose.

A retrospective cohort study at the All-India Institute of Medical Sciences measured the rate of reinfection (tested by RT-PCR or rapid antigen) in BBV152-vaccinated health care workers during the Delta variant surge in India (101). The 1917 health care workers were divided into 3 groups: unvaccinated (n = 472), partially vaccinated (n = 356), and fully vaccinated (n = 1089). Among the unvaccinated group, SARS-CoV-2 reinfection was observed in 60 of 472 (12.7%) with an incidence density (ID) of 18.05 per 100 person-years (95% CI, 14.02-23.25). Reinfection among partially vaccinated individuals was 39 of 356 (11.0%) with an ID of 15.62 per 100 person-years (95% CI, 11.42-21.38), and among fully vaccinated individuals was 17 of 1089 (1.6%) with an ID of 2.18 per 100 person-years (95% CI, 1.35-3.51). The estimated effectiveness of BBV152 against reinfection was 86% (95% CI, 77%-92%) in fully vaccinated individuals, with their IDs of 0.08 (95% CI, 0.01-0.55) and hospitalization rates of 0.13 (95% CI, 0.01-1.28) (101). A similar study was conducted in New York state to assess the effectiveness of the BNT162b2 (Pfizer), mRNA-1273 (Moderna), and Ad26.COV2.S (Johnson & Johnson) vaccines (102). During the week of August 28, 2021, when the Delta variant accounted for 99.6% of all COVID-19 infections in New York state, the median vaccine effectiveness against reinfections was 77.8% (95% CI, 70.1 - 86.8) for mRNA-1273 followed by 72.3% (95% CI, 63.7 - 77.5) for BNT162b2 and 69.4% (95% CI, 63.4 - 77.3) for Ad26.COV2.S. The combined median effectiveness against infection in fully vaccinated people during this Delta surge was 74.2% (95% CI, 63.4 - 86.8) and the effectiveness against hospitalizations was more than 90% (102). Therefore BBV152 showed equivalent protection levels as BNT162b2, mRNA-1273, and Ad26.COV2.S vaccines against breakthrough infections and hospitalizations in fully vaccinated individuals.

Heterologous prime-boost using different vaccine products allows for flexibility in cases of unpredictability or lack of supply of the same vaccine and may enhance vaccine effectiveness by reducing reactogenicity and enhancing the net vaccine effectiveness (103). A retrospective cohort study compared the virus neutralization (NAb) profiles of BBV152 and ChAdOx1-nCov-19 homologous prime-boosted individuals to those that were heterologous prime-boosted (first ChAdOx1-nCov-19 dose followed by second BBV152 dose) by PRNT_50_ assays (104). The geometric mean titer of NAbs against the B.1, Alpha, Beta and Delta variants for BBV152 were 162 (95% CI, 76.74-342), 122.7 (95% CI, 59.36-253.7), 48.43 (95% CI, 19.71-119) and 51.99 (95% CI 19.65-137.6) in the ChAdOx1-nCov-19 group and 156.6 (95% CI, 105.2-233.1), 112.4 (95% CI, 76.56-164.9), 52.09 (95% CI, 34.9-77.73) and 54.37 (95% CI, 27.26-108.4) in the BBV152 group. However, the sera of the heterologous group had roughly 3-fold higher NAb titers of 539.4 (95% CI, 263.9-1103), 396.1 (95% CI, 199.1-788), 151 (95% CI, 80.21-284.3), and 241.2 (95% CI, 74.99-775.9) respectively against B.1, Alpha, Beta and Delta variants (104). They also concluded that the heterologous group had reduced or similar reactogenicity and adverse events. A similar study by Atmar et al. concluded that the vaccination efficacies for Ad26.COV2.S followed by BNT162b2 or mRNA-1273 were 10 to 20-fold higher than Ad26.COV2.S homologous prime-boost vaccination with at par reactogenicity (105). It would be interesting to study if a dissimilar vaccine like BBV152 (the whole virus inactivated) would synergize the efficacies of mRNA vaccines like BNT162b2 or mRNA-1273 in the future heterologous prime-boost studies.

## Impact of a third BBV152 booster dose on the persistence of anti-SARS-CoV-2 immunity

Multiple studies report the decline in anti-SARS-CoV-2 NAb titers within 2-6 months post-infection or immunization (106). The emergence of VOCs as the dominant infectious strains raises further concerns about the durability of vaccine responses since the level of NAb titers protective against the original strain might not be protective against VOCs (**Figure 3**) (107–110). With an increase in breakthrough infections by the highly transmissible Omicron (B.1.1.529) variant, it is vital to understand the role of NAb persistence in long-term vaccine efficacy (102, 111). The impact of a third BBV152 dose was tested on 184 randomly selected candidates of an ongoing BBV152 Phase II trial (ClinicalTrials.gov: NCT04471519). Candidates were divided into placebo (n= 93) or 6 µg BBV152-Algel-IMDG (n=91) groups and the dose was administered on day 215 (approximately 6 months) after the primary dose. Geometric mean NAb titers (PRNT_50_) induced 4 weeks after the second BBV152 dose were 197.0 (95% CI, 155.6–249.4) and reduced to 23.9 (95% CI, 14.0–40.6) by six months. Four weeks after the third (booster) BBV152 dose, the NAb titers increased to 746.6 (95% CI, 514.9–1081) compared with 100.7 (95% CI, 43.6–232.6) in the placebo group with seroconversion rates being 98.7% (95% CI, 92.8–99.9) and 79.8% (95% CI, 69.6–87.8), respectively. The third BBV152 dose led to increased NAb titers against VOCs such as Alpha (32.6-fold), Beta (161-fold), and Delta (264.7-fold). The BBV152 third dose also induced memory B-cells, central and effector memory CD4 T-cells, and cytotoxic effector memory CD8 T_TEMRA_ cells (112). A more recent study compared the effect of the BBV152 booster (third) dose given 215 days after the second BBV152 dose in fully vaccinated individuals. The booster dose recipients had 19.11 (*P* < 0.0001), 14.70 (*P* = 0.0002), 16.51(*P* < 0.0001) and 18.53 (*P* = 0.0002) fold higher NAb titers against B.1, Beta, Delta and Omicron, respectively (113). Thus, the booster dose of Covaxin robustly triggered NAb responses and efficiently neutralized the multiple VOCs of SARS-CoV-2

## BBV152 pediatric study

In 2020 at the onset of the pandemic, in adults, COVID-19 infections in children were not associated with severe symptoms requiring hospitalization or ventilation support, and pediatric deaths due to COVID-19 were limited to children with underlying chronic medical conditions (114, 115). Although asymptomatic during active COVID-19 infections, children have shown similar viral loads as adults and could be the source of ongoing infections (116–119). The new VOC have almost entirely replaced the original SARS-CoV-2 strain leading to higher rates of symptomatic and more severe COVID-19 in children (120). The failure to decrease infection rates in the ongoing pandemic has been associated with an increasing burden of infections in children (121). Therefore, several countries have now considered including children in their COVID-19 vaccination programs (122–124), making it a priority to develop new vaccines or establish the safety of current vaccines in children. A Phase II/III open-label, multi-center age de-escalation study was performed across six hospitals in India, in three pediatric age cohorts (n=526): ≤18->12years (group 1: n=176), ≤12->6 years (group 2: n=175) and ≤6->2 years (group 3: n=175) (125). No severe adverse events were reported with mild to moderate fever being the most frequent systemic events reported after the primary dose (group1- 5%, group2 – 10% & group3 – 13%), decreasing to 4% or less after the booster dose in all groups. Seroconversion rates in all 3 groups were equivalent to those seen in the adult population: group1 = 94.9% (95% CI, 90.5-97.5), group2 = 98.2% (95% CI, 94.9-99.6), and group 3 = 98.3% (95% CI, 95.0-99.6). All three pediatric groups showed 2-fold higher titers of NAbs (by PRNT_50_) than adults and IgG1:IgG4 ratios > 1 indicative of a Th1-biased robust anti-SARS-CoV-2 immune response (125). Both vaccine and placebo reactogenicity were similar and no severe adverse events were reported. Based on these findings BBV152 received emergency use listing in India, for children 11-18 years old in December 2021 and for children 6-11 years old in April 2022.

## Conclusions

BBV152 is an exciting player in the Anti-SARS-CoV-2 vaccination landscape. It is a whole-virion inactivated SARS-CoV-2 vaccine generated against the B.1.1.7 variant and administered with the TLR7/8 agonist molecule – Algel-IMDG, to induce a Th1-biased immune response. In contrast to mRNA or viral vectored vaccines which express the Spike protein as the only antigen target, inactivated - purified BBV152 particles show intact coronaviral morphology and induce antibody responses against both the Spike and the Nucleocapsid proteins. BBV152 was safe in preclinical animal models such as BALB/c mice, Wistar rats, New Zealand rabbits, Syrian hamsters, and rhesus macaques, and generated protective NAbs plus CD4/CD8 T-cell responses (66, 74, 75). BBV152-vaccinated animals not only cleared the viral challenge but were also protected from COVID-19 pneumonia and associated irreversible damage to other organs. The BBV152 Phase I, II, and III trials established not only its safety in humans but also its efficacy at engaging CD4 and CD8 T-cell responses in addition to producing protective NAb titers. BBV152 protected up to 78% of vaccinated candidates against symptomatic infection, 93.4% from severe disease, and 63.6% from asymptomatic disease (56). Additionally, sera from BBV152 vaccinated candidates in Phase I/II clinical trials could neutralize all five VOCs as measured by PRNT_50_ assays. However, as reported with other vaccine candidates this neutralization efficacy was reduced with Delta and Omicron variants (91–94).

Studies show that SARS-CoV-2 vaccinations at $35 per dose even at 60% efficacy are considerably cost effective ($8,200 per quality adjusted life-year (126). As the highly transmissible Omicron (B.1.1.529) variant becomes dominant globally, the protective NAb titers from initial vaccination are proving to be inadequate in preventing breakthrough infections (102, 111). A third BBV152 booster, 6 months after the prime vaccine dose, not only reverses the decline in the NAb titers with time but also leads to a significant increase in NAb titers which might provide better protection against multiple VOC especially the Omicron variant (113). The BBV152 third dose also induced memory B-cells, central and effector memory CD4 T-cells, and cytotoxic effector memory CD8 T_TEMRA_ cells (112). Perhaps one of the most interesting pieces of data to emerge from the coordinated global vaccination efforts is the possibility to enhance the immune response by heterologous prime-boost vaccination. The WHO Strategic Advisory Group of Experts on Immunization (SAGE) reviewed the evidence and issued the following interim guidance: Although the direct evidence is limited due to the multiplicity of different vaccine combinations, heterologous schedules have consistently shown enhanced immunogenicity when inactivated virus vaccines were administered either before or after vectored or mRNA vaccines (103, 104, 127). Certain key gaps in the evidence to be addressed include: 1) effectiveness and duration of protection of heterologous versus homologous vaccine schedules, especially for heterologous schedules involving inactivated vaccines; 2) long-term safety, immunogenicity, and effectiveness of heterologous vaccination, and surveillance for rare adverse events; 3) the ideal time interval between the primary series and booster dose; 4) correlates and duration of protection for homologous and heterologous schedules; and 5) immunogenicity and efficacy of heterologous and homologous vaccine schedules against VOC like the Omicron variant.

## Challenges and the path forward

**Figure 4:**
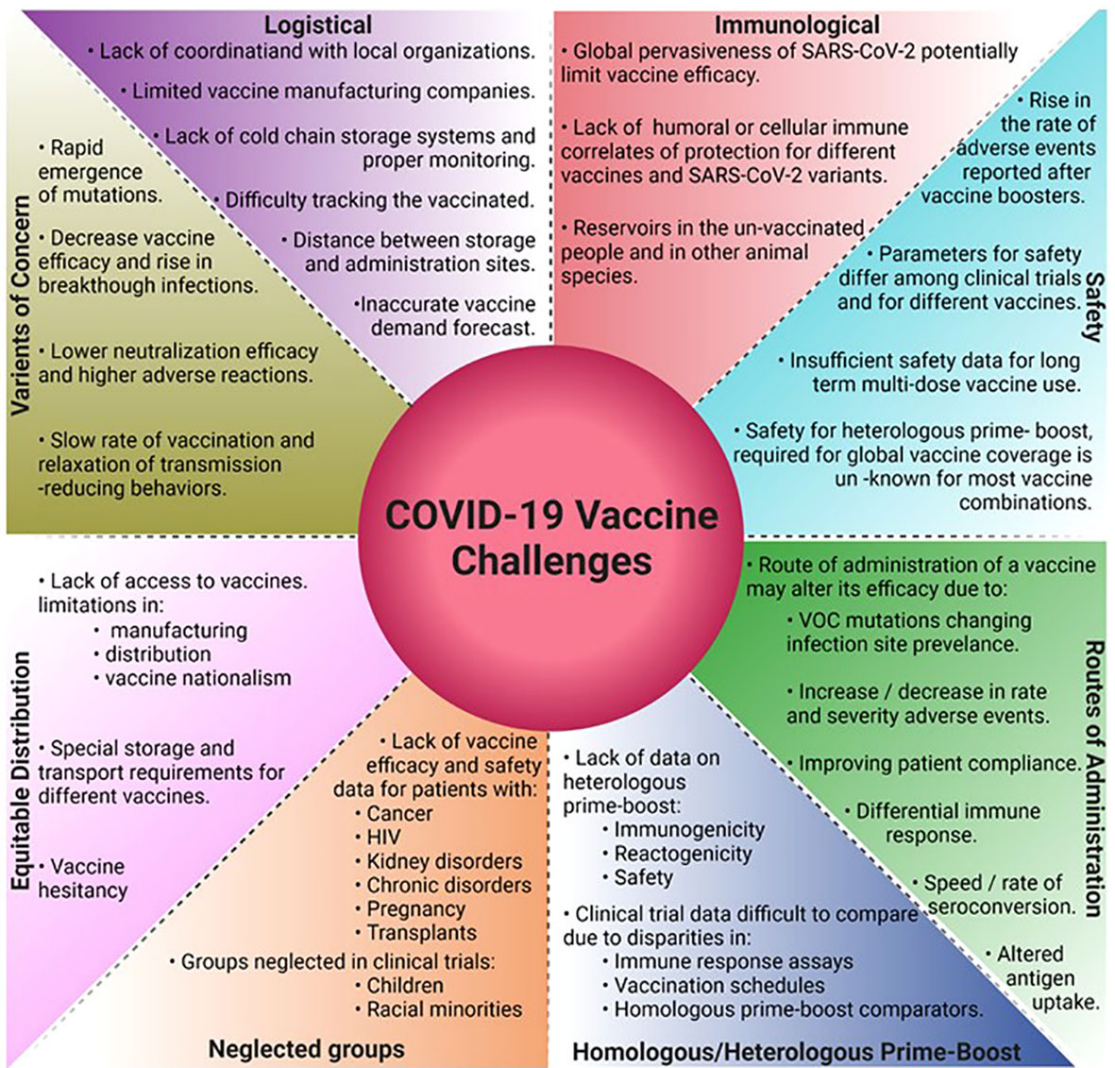
Challenges in the development and deployment of COVID-19 vaccines, and the path forward.

The global vaccine campaigns, a testament to human innovation and dedication have encountered and overcome several challenges ranging from scientific, logistical, societal, and marketing (**Figure 4**). Key implementation parameters like the manufacturing and distribution speed and the extent of vaccine delivery in a population plays just as critical a role in vaccination success as the efficacy of the vaccine itself. In the US, Operation Warp Speed was critical for early vaccination efforts by infusing investments in vaccine development, manufacturing, and distribution, however reaching vaccine-hesitant and underserved groups limited the success of the vaccination program. Globally in underserved areas, efforts to design and implement strategies on supply chain and distribution are still desperately needed (128).

Almost all SARS-CoV-2 vaccines being developed require prime-boost vaccination with protective efficacies declining by 6 months. The global vaccination endeavors face additional challenges from the emergence of VOCs that might require higher NAb titers for protection. To fulfill the global demand would therefore require pharma companies to supply billions of doses annually and the governments to develop effective prioritization strategies. Nucleic acid and viral vector vaccines that require storage at −70°C from manufacturing to vaccination run into ultra-cold chain issues for distribution in rural and underdeveloped areas that limit their use. BBV152 can be stored and distributed at 2 to 8°C using existing cold chain infrastructure to mitigate these issues (129). There is a lack of well-defined correlates of protection against SARS-CoV-2 infection such as the exact protective antibody type and titer levels, T-cell responses responsible for the range of symptoms, and the variation in these correlates concerning VOCs. Knowing this information would provide measurable aspects of immune responses required to prevent severe symptoms, decrease breakthrough infections, and help develop vaccines that confer sterilizing immunity and prevent infection rather than just prevent disease. Additional consideration must be given to safety and efficacy trials in children, pregnant women, and groups with severely compromised immunity such as HIV+ and cancer patients under treatment (130–132). Currently, all leading vaccines are administered through the intramuscular route. However, several studies report the importance of mucosal immunity against SARS-CoV-2 infections (133–136), and 12 intranasal vaccine candidates are currently in different clinical phases around the world (137). The Omicron variant, currently the most dominant global SARS-CoV-2 variant, shows enhanced viral replication in the human bronchus and upper respiratory tract than the lungs (138). Although Omicron shows reduced damage to the lungs and therefore reduced disease severity, its enhanced transmission capacity poses a major threat to public health worldwide. The increased Omicron-related breakthrough infections in the vaccinated population could in part be due to poor or no mucosal immunity generated by the intra-muscular vaccines. Therefore, the role of booster doses with intranasal vaccines needs to be investigated further. Real-world data on the efficacy and safety of multiple homologous booster vaccines are just emerging. Of particular concern is the lowered efficacy of the 4^th^ mRNA vaccine booster dose against the Omicron variant (BNT162b2 -30% and mRNA1273 -11%) and the rise in vaccine-related adverse events (48). As recent history suggests, emerging VOCs have been more transmissible than their previous counterparts with Omicron being about 3-fold or 300% more transmissible than Delta. Next-gen vaccines may be needed to improve the neutralization efficacy against future VOC and will take time to develop. While some studies report improvements in vaccine efficacy by heterologous prime-boost regimens (103–105), a more robust clinical study on the immunogenicity, reactogenicity, and safety of multiple vaccine combinations and schedules is required. One such study underway in the US will evaluate the immunogenicity and safety of BBV152 booster dose in participants fully vaccinated (two doses) with mRNA vaccines (ClinicalTrials.gov: NCT05258669).

## Author contributions

All authors made substantial contributions in the article preparation at the following stages: (1) the conception and design of the manuscript and interpretation of data, (2) drafting and revising the article for important intellectual content, (3) final approval of the version to be submitted.

## Funding

This research did not receive specific funding but was performed as part of the employment of the authors, by Ocugen Inc.

## Conflict of interest

The authors are employed by Ocugen Inc., which has commercialization rights for BBV152 in North America.

## Publisher’s note

All claims expressed in this article are solely those of the authors and do not necessarily represent those of their affiliated organizations, or those of the publisher, the editors and the reviewers. Any product that may be evaluated in this article, or claim that may be made by its manufacturer, is not guaranteed or endorsed by the publisher.
